# Elastic shape analysis of brain structures for predictive modeling of PTSD

**DOI:** 10.3389/fnins.2022.954055

**Published:** 2022-09-01

**Authors:** Yuexuan Wu, Suprateek Kundu, Jennifer S. Stevens, Negar Fani, Anuj Srivastava

**Affiliations:** ^1^Department of Statistics, Florida State University, Tallahassee, FL, United States; ^2^Department of Biostatistics, The University of Texas MD Anderson Cancer Center, Houston, TX, United States; ^3^Department of Psychiatry and Behavioral Sciences, Emory University, Atlanta, GA, United States

**Keywords:** computational anatomy, elastic shape analysis, PTSD diagnosis, statistical regression models, shape PCA

## Abstract

It is well-known that morphological features in the brain undergo changes due to traumatic events and associated disorders such as post-traumatic stress disorder (PTSD). However, existing approaches typically offer group-level comparisons, and there are limited predictive approaches for modeling behavioral outcomes based on brain shape features that can account for heterogeneity in PTSD, which is of paramount interest. We propose a comprehensive shape analysis framework representing brain sub-structures, such as the hippocampus, amygdala, and putamen, as parameterized surfaces and quantifying their shape differences using an elastic shape metric. Under this metric, we compute shape summaries (mean, covariance, PCA) of brain sub-structures and represent individual brain shapes by their principal scores under a shape-PCA basis. These representations are rich enough to allow visualizations of full 3D structures and help understand localized changes. In order to validate the elastic shape analysis, we use the principal components (PCs) to reconstruct the brain structures and perform further evaluation by performing a regression analysis to model PTSD and trauma severity using the brain shapes represented *via* PCs and in conjunction with auxiliary exposure variables. We apply our method to data from the Grady Trauma Project (GTP), where the goal is to predict clinical measures of PTSD. The framework seamlessly integrates accurate morphological features and other clinical covariates to yield superior predictive performance when modeling PTSD outcomes. Compared to vertex-wise analysis and other widely applied shape analysis methods, the elastic shape analysis approach results in considerably higher reconstruction accuracy for the brain shape and reveals significantly greater predictive power. It also helps identify local deformations in brain shapes associated with PTSD severity.

## 1. Introduction

Scientific studies have increasingly documented the extent of traumatic experiences in our society—60.7% of men and 51.2% of women would experience at least one potentially traumatic event (Javidi and Yadollahie, 2012). A significant proportion of these events occurs during young age; for example, 26% of males and 18% of females reported having experienced at least one traumatic event at a young age (Perkonigg et al., 2000). Of those experiencing traumatic events, 15–40% develop psychiatric symptoms of clinical relevance (Priebe et al., 2010), such as post-traumatic stress disorder (PTSD). PTSD is the fourth most common mental disorder in the USA and results in significant impairments of psychological and physical health (Kessler et al., 1995; Sareen, 2014). Diagnosing psychiatric disorders using brain imaging data has started gaining attention in the literature. Imaging provides physical biomarkers, such as volumes and shapes of anatomical structures in the human brain. Subcortical structures, in particular, have been implicated in preclinical neuroscience research in behaviors and phenotypes with crucial relevance to mental health.

Voxel based morphometry (VBM) approaches have identified associations between PTSD and the volumes of a variety of brain regions including the amygdala, prefrontal cortex, temporal cortex, insula, thalamus, anterior cingulate cortex (ACC), and hippocampus (Nemeroff et al., 2006; Francati et al., 2007; Mahan and Ressler, 2012). All of these regions contain sub-regions with specialization in function, and likely different links to psychopathology. Sub-regions have been investigated in PTSD using automated segmentation methods (Van Leemput et al., 2009; Saygin et al., 2017). For example, PTSD-related alterations in the hippocampus have been isolated to lower volume of the CA1 sub-field (Chen et al., 2018). Findings are more mixed for amygdalar subnuclei. In adult military veterans, PTSD is linked with smaller paralaminar and lateral subnuclei, but larger central, medial, and cortical nuclei which are critical to the behavioral and physiological outputs of fear (Morey et al., 2020). In contrast, in youth exposed to a terror attack, PTSD symptoms were associated with smaller volumes across all major subnuclei (Ousdal et al., 2020). These findings suggest that the developmental timing and type of trauma exposure may have important effects on neural phenotypes in PTSD. However, the analyses of subnuclei are limited by the limits in tissue contrast and spatial resolution available in typical 3T MRI research scans. Alternative methods for understanding the morphology of subcortical regions are likely to provide important neural biomarkers of various psychiatric conditions.

There is limited literature on brain shape changes in PTSD, although the relationship between morphologies of structures such as hippocampus and putamen shapes with disorders such as ADHD, Alzheimer's, and Schizophrenia are well-established (Kurtek et al., 2011b; Joshi et al., 2016). The value of brain shape analysis lies in its ability to reveal local regions of variation within a structure's surface. This is valuable complementary information to the volumetric descriptions of a structure which depict gross variation in a single direction (i.e., increased or decreased volume). The addition of localized topology descriptions allows the detection of subtler changes in the morphometry of a surface whose signal may be lost when averaged across the whole ROI. For example, although hippocampal changes are expected between PTSD and control groups, volumetric analyses in (Veer et al., 2015; Bae et al., 2020) found that the differences in bilateral hippocampus were not significant. In contrast, full shape analysis can allow investigators to detect regions of equal but opposite variation within a single surface, which would have otherwise been canceled out had they been reduced to a single scalar value. Furthermore, despite the recent developments of limited literature concerning the use of brain structures for assessing PTSD severity, there are significant unanswered questions. They related to *how* alterations in the brain structure and shape after trauma exposure results in PTSD onset and progression.

The most commonly used tools for shape analysis in PTSD involve an FSL toolbox pipeline that is utilized to identify the correlation between PTSD and subcortical volumes and shapes, most focused on the amygdala and hippocampus (Veer et al., 2015; Akiki et al., 2017; Knight et al., 2017; Klaming et al., 2019; Bae et al., 2020). This pipeline, known as FIRST, is a surface-based vertex-wise shape analysis (Patenaude et al., 2011) method that compares the brain surface distances between the PTSD and control populations *via* multivariate statistics such as radial distance and Jacobian determinant. Other methods (Tate et al., 2016) have used spherical shape registration tools proposed in Gutman et al. (2015). The latter uses a combination of spherical and medial axis representations to achieve a final surface registration. Although such analyses are useful, they essentially rely on a vertex-wise analysis that visualizes the brain surface as a collection of discrete vertices represented by voxels, which overlooks the interpretation of the brain shape as a continuously varying object in three dimensions. Moreover, due to a large number of voxels included in the multivariable analysis, it is challenging to accommodate interactions between the brain shape and confounding variables such as trauma exposure without giving rise to an inflated number of parameters in the model. Existing approaches in Knight et al. (2017) and Klaming et al. (2019) overcome this difficulty by including interactions *via* a voxel-wise analysis, which ignores the spatial nature of the brain shape, and requires stringent multiplicity adjustments for testing significant effects. These limitations are likely to result in biological findings that may not be reproducible across studies, especially in studies with moderate sample sizes. For example, the vertex-wise analysis in Knight et al. (2017) did not find significant subcortical volume or shape differences between PTSD and control groups, although there were weakly significant interaction effects between depression and PTSD severity in the left amygdala. In contrast, Veer et al. (2015) found smaller right amygdala volume for PTSD vs. control, while Bae et al. (2020) discovered larger left amygdala volume for PTSD vs. control group.

In this article, we apply *elastic shape analysis* method (Jermyn et al., 2017) to analyze brain subcortical structures. It characterizes shapes as parameterized surfaces in ℝ^3^, instead of point sets, and it incorporates dense registration of points across objects. The elastic shape analysis quantifies pure shape variability, modulo shape-preserving transformations, and helps discern subtle variations across populations by minimizing mis-registration errors. The elastic shape analysis helps align, register, and compare shapes of surfaces. It also provides shape summaries, i.e., statistical means and covariances of shapes sampled from a population. The computation of covariance leads to principal component analysis (PCA) of sample shapes and the set of principal coefficients (PC) forms a parsimonious, low-dimensional representation of those brain structures. By including interactions between a subset of PCs and confounding variables (such as trauma), the proposed approach provides a parsimonious and flexible classification or prediction approach not restricted to vertex-wise analysis. It enables non-linear associations between the brain shape, potential confounders, and their interactions with the clinical phenotype of interest. This results in superior prediction performance compared to the widely applied shape analysis methods in PTSD literature.

An essential strength of these shape features is reconstructing full shapes from these feature vectors (principal components). Consequently, one can visualize changes in the shapes of a structure by varying only one or multiple features at a time. This provides a vital tool for physicians and clinicians to visualize localized changes or deformations in the brain anatomy for statistically significant shape features or principal components. We focus on a pre-specified subset of subcortical structures and their interactions with demographic and exposure confounding variables to classify and predict PTSD severity. In contrast to most existing studies that focus on brain shape changes in military veterans, our study is one of the first to investigate brain shape changes in PTSD in conjunction with co-morbidities such as trauma in a civilian minority population of AA females.

The main contributions of this paper are:

1. The use of elastic shape analysis to characterize shapes of subcortical structures as parameterized surfaces. This framework integrates the registration of surfaces as a part of shape analysis, and provides a comprehensive toolbox for registering, comparing, summarizing, and testing shapes. This leads to a representation of shapes using (invertible) PCA features. Many of the past works that utilize anatomical shapes in medical diagnoses represent shapes as point clouds, i.e., a set of discrete points. In contrast, we follow an approach that incorporates full surface geometries of anatomical objects, which results in more accurate reconstructions and considerably more accurate predictions.
2. The use of shape features and other clinical covariates in statistical regression models for modeling PTSD severity measures as response variables. This results in shortlisting and analysis of features that are significant in predicting PTSD. Furthermore, it allows us to include the interaction terms in the regression models also. While elastic shape distances have been used for predicting clinical measures in some previous works (Kurtek et al., 2011a; Joshi et al., 2016), the use of full elastic shapes has not been explored and presents a new methodological contribution of independent interest.
3. The visualization of local deformations associated with significant morphological features and their interpretations in predicting PTSD severity. A significant outcome of this framework is these tools that allow physicians and other experts to validate the findings through visualizations, thus making it easier to incorporate into clinical practice.

## 2. Materials and methods

**Figure 1:**
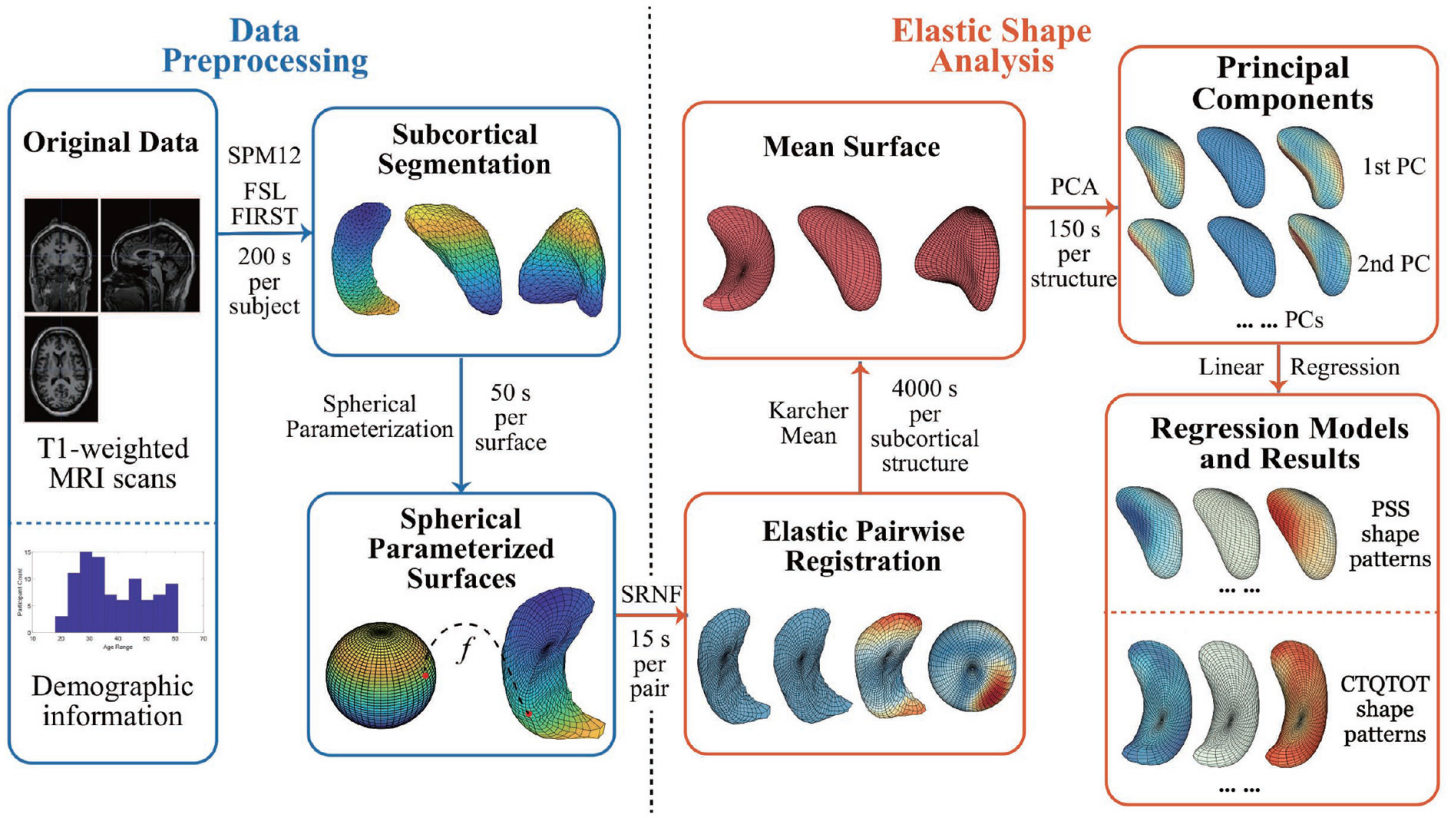
Pipeline steps and time cost. The time cost is computed with Intel i7-8705G. SRNF, square root normal field; PCA, principal component analysis; PC, principal component; PSS, PTSD symptom scale; CTQTOT, childhood trauma questionnaire total score.

This section lays out the entire pipeline for extracting and analyzing shapes of subcortical brain surfaces. This pipeline is illustrated pictorially in Figure 1 with the time costs computed on a laptop with Intel i7-8705G processor. As the figure shows, the proposed pipeline has three main steps: (i) pre-processing of the original data; (ii) registration and shape analysis of 3D surfaces; and (iii) regression models for analysis of PTSD. We describe these steps next, starting with introducing the data used in the experiments presented later.

### 2.1. Data description

In this study, we utilize T1-weighted MRI scans of brains of 90 subjects. The dataset also contains demographic information with the questionnaire results on PTSD symptoms and traumatic experiences.

#### 2.1.1. T1-weighted MRI scans

The original data is T1-weighted MRI scans acquired by Emory University Grady Trauma Project using Siemens Tim Trio (Logue et al., 2018). Field of view is 224 × 256 mm, while repetition time and echo time are 2,600 and 3.02 ms separately.

#### 2.1.2. Demographic information

Participants of the experimental data collection are all African American women. The other demographic information included in the data is: age (18–61), education (0–5), employment (0,1), and disability (0,1).

#### 2.1.3. Questionnaire results

In the study, we are most interested in participants' answers to questions related to PTSD symptoms and traumatic experiences. Specifically, we focus on three questionnaire results: (i) PTSD Symptom Scale (PSS), which measures the presence and frequency of current PTSD symptoms and has a range of 0–42; (ii) Childhood Trauma Questionnaire Total Score (CTQTOT), which is a 25-item inventory of different types of childhood maltreatment including abuse and neglect and has a range of 25–125; and (iii) Beck Depression Inventory (BDI), which is a 21-question multiple-choice self-report inventory and has a range of 0–63.

### 2.2. Data pre-processing

Here we describe the steps for extracting subcortical structures from brain imaging data. We use the widespread packages to pre-process the MRI scans obtained as the original data. We first convert 176 DICOM scan files for each subject into a single NIfTI file using SPM12 (Penny et al., 2011). The NIfTI images each have a resolution of 240 × 256 × 176.

Next, we utilize the FMRIB Software Library (FSL) that contains image analysis and statistical tools for functional, structural and diffusion MRI brain imaging data. Among the tools in FSL, FSL FIRST (Patenaude et al., 2011) is a model-based segmentation/registration tool. FSL FIRST can segment a T1-weighted MRI image into 15 subcortical structures' surfaces. Using some manually segmented images, in which the subcortical labels are parameterized as surface meshes and modeled as a point distribution model, FSL FIRST trains an automatic segmentation model using a Bayesian approach. The inputs of FSL FIRST are T1-weighted MRI images in NIfTI file format, and the outputs are triangular meshed surfaces of 15 subcortical brain structures. Although there are several structures available for study, this paper mainly focuses on three structures: left hippocampus, left amygdala, and left putamen. They are identified as the most related subcortical structures in the existing literature (Filipovic et al., 2011; Veer et al., 2015; Zhong et al., 2015; Akiki et al., 2017; Knight et al., 2017; Klaming et al., 2019; Bae et al., 2020).

**Figure 2:**
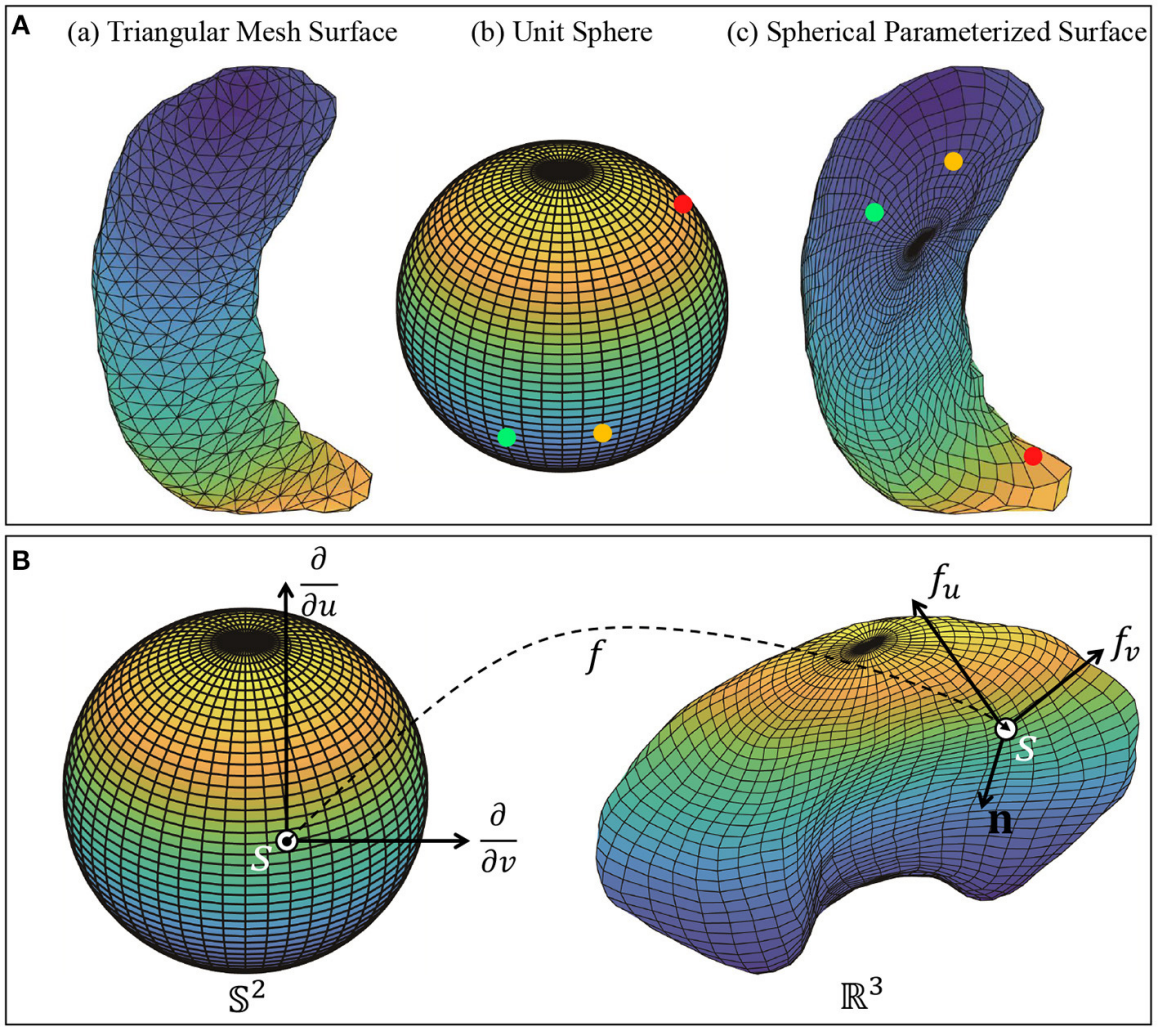
**(A)** Object pre-processing: Triangular mesh surface and spherical parameterized surface. Each vertex on the spherical parameterized surface corresponds to a vertex on the meshed unit sphere *S*^2^. **(B)** Local geometry of a parameterized surface viewed as a mapping *f*:𝕊^2^ → ℝ^3^. The figure shows the tangent vectors *f*_*v*_ and *f*_*u*_ and the normal vector **n** at point *f*(*s*) on *f*.

We then apply a spherical conformal mapping and Tuette mapping algorithms in Jermyn et al. (2017) to transform the triangulated meshes into *spherically-parameterized* surfaces. The method first creates progressively finer mesh structure with triangles, and then embeds the mesh vertices into a sphere. The surfaces are spherically parameterized since each point on the surface corresponds uniquely to a point on the unit sphere *S*^2^. This provides a representation of the surface as an embedding: *f*:*S*^2^ → ℝ^3^. Figure 2A illustrates the process: Column (a) shows an example of triangular mesh surface, column (b) and (c) are sphere *S*^2^ and the corresponding spherically-parameterized surface. Points in the same color indicate the corresponding relationship.

### 2.3. Framework: Elastic shape analysis

To analyze shapes of subcortical structures and to discern shape changes with traumatic experiences, we utilize the *elastic shape analysis* approach, developed in the book (Jermyn et al., 2017). This comprehensive theory provides several tools for analyzing shapes of 3D objects, including (1) metric for quantifying differences in their shapes, (2) deforming objects into each other using geodesic paths, (3) optimally registering points across surfaces being compared, and (4) computing mean, covariance, and PCA of shapes. An important aspect of this approach is that these results are theoretically invariant to the chosen shape-preserving transformations (rigid motions, global scaling, and parameterizations of surfaces). From a practical perspective, it helps remove misregistration errors from the analysis. In the past, this framework has been applied to shape analysis of human bodies (Laga et al., 2017) and brain morphology associated with Alzheimer's (Joshi et al., 2016) and ADHD (Kurtek et al., 2011a).

#### 2.3.1. Elastic metric and SRNF

Next, we present some salient ideas of this approach. The subcortical objects are considered as closed surfaces in ℝ^3^. Each closed surface can be represented in a parameterized form using a smooth map: *f*:*S*^2^ → ℝ^3^. Let F denotes the space of all such surfaces. If *s* = (*u, v*) is a point on the sphere *S*^2^, then the partial derivatives *f*_*u*_ and *f*_*v*_ denote two orthogonal tangent vectors to the surface *f* at the point *f*(*s*). The (unnormalized) normal vector at point *s* is given by **n**(*s*) = *f*_*u*_ × *f*_*v*_, where × indicates the cross product in ℝ^3^. Figure 2B shows a surface *f*, parameterized by points on a unit sphere *S*^2^, with the tangent and normal vectors at point *f*(*s*) on the surface.

Let Γ be the set of all orientation-preserving diffeomorphisms of *S*^2^; the elements of Γ help us re-parameterize surfaces. For any parameterized surface f∈F and a γ∈Γ, the composition *f*°γ denotes a re-parameterization of *f*. Equivalently, elements of γ also help in a dense registration of points across two surfaces, say *f*_1_ and *f*_2_. Initially, for any *s*∈*S*^2^, the point *f*_1_(*s*) on *f*_1_ is said to be registered to the point *f*_2_(*s*) on *f*_2_. However, if we re-parameterize *f*_2_ by γ, then the point *f*_1_(*s*) is now registered to the point *f*_2_(γ(*s*)) on *f*_2_. Thus, γ becomes a tool for controlling the registration between *f*_1_ and *f*_2_. The next question is: How can we find the best registration between any two surfaces *f*_1_ and *f*_2_? A related question is: What should be the objective function for defining and calculating the optimal γ that best registers *f*_2_ with *f*_1_? An obvious choice would be the *L*2 norm, but it is degenerate and leads to singularities in solutions. While one can impose additional penalties to avoid degeneracy, the resulting solution is not inverse symmetric. That is, the registration of surface *f*_1_ to *f*_2_ may not be consistent with the registration of surface *f*_2_ to *f*_1_. From a mathematical perspective, the problems in using the *L*2 norm for registering surfaces stem from the following fact. In general, for any $f_{1},f_{2}\in F$ and γ∈Γ, we have: ||*f*_1_−*f*_2_||≠||*f*_1_°γ−*f*_2_°γ||. In the other words, we lose some information about the shape of the surfaces after re-parameterization if we use the *L*^2^ distance to compare them. A better alternative for registration and shape analysis comes from an elastic Riemannian metric. While this metric's original form is too complex for practical usage, a square-root representation of surfaces simplifies their usage. This representation, termed the square root normal field (SRNF), is defined as follows: for *s*∈*S*^2^, define $q\left(s\right)=\text{n}\left(s\right)/|\text{n}\left(s\right){|}^{\frac{1}{2}},$ where **n**(*s*) is the normal at a point *f*(*s*) as explained earlier. Thus, *q* is nothing a but a normal vector field on the surface *f* with the magnitude given by $\sqrt{\left|\text{n}\left(s\right)\right|}$. SRNF of the re-parameterized surface *f*°γ is given by $\left(q◦\gamma \right)\sqrt{{\text{J}}_{\gamma }}$, where **J**_γ_ is the determinant of the Jacobian of γ.

The most important property of elastic shape analysis is that: for any two surfaces $f_{1},f_{2}\in F$ and their SRNFs *q*_1_, *q*_2_, we have the famous **invariance property**:


(1) $$\begin{array}{r} \left||q_{1}-q_{2}||=||O\left(q_{1}◦\gamma \right)\sqrt{{\text{J}}_{\gamma }}-O\left(q_{2}◦\gamma \right)\sqrt{{\text{J}}_{\gamma }}|\right| \end{array}$$

for all 3D rotations *O*∈*SO*(3) and all γ∈Γ. Such an invariance is not present in any other method that has been discussed in the paper. This is an important fundamental limitation of non-elastic, non-Riemannian approaches.

Let $C=L^{2}\left(S^{2},{\mathbb{R}}^{3}\right)$ be the pre-shape space of all SRNFs. Then, due to the invariance property (1), we can define a proper metric on the shape space C/(Γ×SO(3)):


(2) $$\begin{array}{r} d_{s}\left(\left[q_{1}\right],\left[q_{2}\right]\right)=\underset{\left(O,\gamma \right)\in SO\left(3\right)\times \Gamma }{\inf}||q_{1}-O\left(q_{2}◦\gamma \right)\sqrt{{\text{J}}_{\gamma }}||\;. \end{array}$$

With this metric we can define a statistical mean and register individual surfaces to this mean according to:


(3) $$\begin{array}{r} \left[q_{\mu }\right]=\arg\underset{\left[q\right]\in C/\left(\Gamma \times SO\left(3\right)\right)}{\min}\overset{n}{\underset{i=1}{\sum }}d_{s}{\left(\left[q\right],\left[q_{i}\right]\right)}^{2}\\ =\arg\underset{\left[q\right]\in C/\left(\Gamma \times SO\left(3\right)\right)}{\min}\\ \;\overset{n}{\underset{i=1}{\sum }}\left(\underset{\left(O_{i},\gamma_{i}\right)\in SO\left(3\right)\times \Gamma }{\inf}||q-O_{i}\left(q_{i}◦\gamma_{i}\right)\sqrt{{\text{J}}_{\gamma_{i}}}|{|}^{2}\right). \end{array}$$

This formula not only defines the mean of given shapes but also describes the registration of each *q*_*i*_ to the mean *q*_μ_. It explains how the shape metric *d*_*s*_ (which is based on pairwise registration) leads to a registration of multiple samples from a population. We refer the reader to two textbooks (Jermyn et al., 2012; Srivastava and Klassen, 2016) for a more detailed explanation of these ideas.

Methods that do not employ proper metrics do not have a well-defined notion of the statistical means and need to provide some separate notion of a “template” for registration. In elastic Riemannian approaches, the template is given by the statistical mean of shapes.

The computation of shape metric in (2) requires solving for the optimal O^*^∈*SO*(3) and γ^*^∈Γ. Before solving for the optimal rotation and re-parameterization, we remove the shape-preserving transformations including translation and global scaling. It is easy to remove these shape-preserving transformations, contributing to the advantage of using the SRNF representation described in the previous paragraphs. Specifically, the SRNFs of surfaces are already invariant to translation. Scaling variability was removed by re-scaling all surfaces to have a unit area: $f=f/\sqrt{\alpha_{f}}$, where $\alpha_{f}=\underset{S^{2}}{\int }|n_{f}\left(s\right)|ds$ is the area of surface *f*. Then, we use the Procrustes method to solve for the optimal rotations and we use a gradient-descent approach to optimize over Γ, whose details and algorithms are presented in Jermyn et al. (2017). The gradient-descent is preceded by a course search over 60 elements of the dodecahedron group to try 60 different rigid rotations (corresponding to 60 placements of the north-south pole coordinate system) and select the minimum. The minimum allows us to get closer to a global solution, and we use that minimum as an initial condition for a gradient search method to find the optimal parameterization. Due to the lack of symmetry in the shapes of these subcortical structures, we expect to get a unique global solution to the registration problem (optimizing over Γ).

#### 2.3.2. Elastic registration: A simulation study

Here, in order to illustrate and validate the necessity of surface registration, we conduct some simulation studies. We randomly generate 40 simulated surfaces using PCA representations of shapes of left hippocampus (the use of PCA is detailed later in this paper). More specifically, we use only the first principal direction *v*_1_ in this experiment and generate 20 surfaces each on either side of mean μ along that direction. That is, we generate *f*_*i*_ = μ+*x*_*i*_*v*_1_, *i* = 1, 2, …, 40, where *x*_*i*_∈(0, 1] for *i* ≤ 20 and *x*_*i*_∈[−1, 0) for *i*>20.

**Figure 3:**
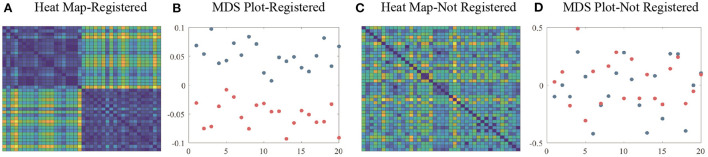
**(A)** Heat map and **(B)** MDS plot of distances between well-aligned and registered surfaces. **(C)** Heat map and **(D)** MDS plot of distances between randomly-parameterized surfaces (not well-registered). Colors of the heat map indicates the relative distances between surfaces. Blue dots in MDS plot indicate the first 20 simulation surfaces and red dots indicate the last 20 simulation surfaces.

Since PCA is performed after surface registration, these simulated surfaces can be considered well aligned and registered. We calculate the pairwise distances between surfaces as *d*_*ij*_ = ||*f*_*i*_−*f*_*j*_||, where *i, j* = 1, 2, ..., 40. Figures 3A,B show the heat map and multidimensional scaling (MDS) plot of the distances between surfaces. First 20 surfaces are presented by blue dots and last 20 are presented by red dots. This figure illustrates that for registered surfaces, the shapes that are on the same direction of principal shape component have relatively small distance, and are correctly clustered into the same class.

Next, we introduce random parameterization functions γ_*i*_∈Γ and apply γ_*i*_'s to *f*_*i*_'s to simulate randomly parameterized surfaces. For each *i*, the surface ${\tilde{f}}_{i}=f_{i}◦\gamma_{i}$ has the same shape as *f*_*i*_, but a different parameterization. The distances between unregistered surfaces are again calculated by ${\tilde{d}}_{ij}=||{\tilde{f}}_{i}-{\tilde{f}}_{j}||$. Figures 3C,D shows the heat map and MDS plot of the distances between randomly parameterized surfaces. We see that distances between surfaces that have similar shapes are not smaller anymore and MDS plot shows that unregistered surfaces are not effectively clustered.

#### 2.3.3. Shape analysis tools: Geodesics, mean, and PCA

The framework developed so far allows for representing and registering anatomical surfaces and comparing shapes of these surfaces pairwise using a proper shape metric. This metric is used to develop some additional statistical tools, leading to a compact way of representing shapes. These tools include finding geodesics between shapes, computing means of shapes of surfaces, and discovering principal modes of shape variation in a given set of shapes.

##### 2.3.3.1. Shape geodesic

**Figure 4:**
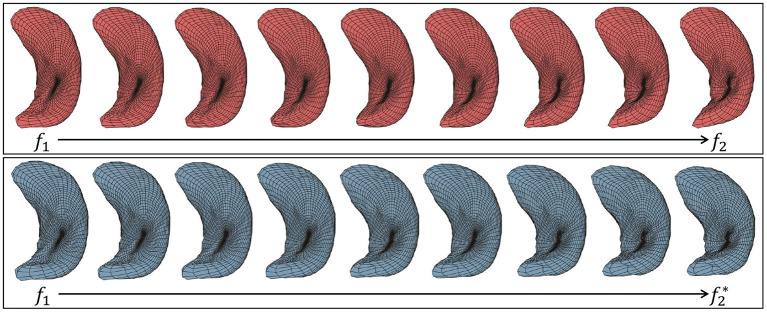
Two examples of geodesic between left hippocampus surfaces. **(Top)** Between unregistered surfaces. **(Bottom)** Between elastic registered surfaces.

Given two surfaces, *f*_1_ and *f*_2_, a geodesic between their shapes is a visualization of the optimal deformation from one to the other. Although there are more sophisticated ways to compute exact geodesics, we use a simple linear interpolation to approximate this deformation according to: $\alpha_{\tau }^{*}\left(s\right)=\left(1-\tau \right)f_{1}\left(s\right)+\tau f_{2}^{*}\left(s\right),\;s\in S^{2}$, where τ∈[0, 1] is the time index of the geodesic and $f_{2}^{*}$ is the optimally registered version of *f*_2_. At τ = 0 we have α = *f*_1_ and at τ = 1 we have $\alpha =f_{2}^{*}$. Figure 4 shows two examples of geodesics, where the upper one is the geodesic between unregistered surfaces and the lower one is between elastically registered surfaces. Due to the misalignment of points between surfaces, the hippocampus's posterior end “degenerates” on the upper geodesic at the midway point. In contrast, the anatomical features of hippocampus surfaces are preserved with elastic registration, making the midway surfaces along the geodesic more interpretable.

Additional examples of elastic geodesics can be found in Supplementary Figure 1. We also provide these deformations as GIF files in the Supplementary material.

##### 2.3.3.2. Mean shape

Here, we introduce the algorithm of computing the mean shape of surfaces defined in (3). We use an iterative algorithm to compute this mean shape. Here we start by selecting an arbitrary surface as the initial guess for μ. Then, in each iteration, we register each *f*_*i*_ with the current mean and compute the (Euclidean) mean of these registered *f*_*i*_'s in F. Once the algorithm has converged, we obtain the mean shape μ. We outline these steps using Algorithm 1.

**Algorithm 1:**
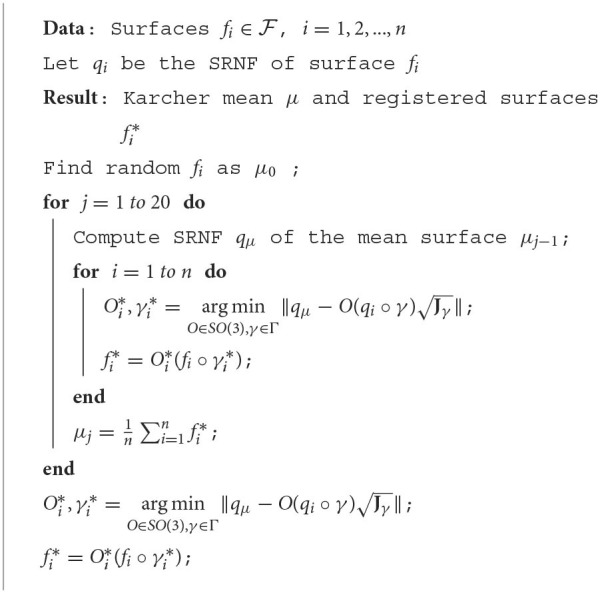
Computing Karcher mean of surfaces.

**Figure 5:**
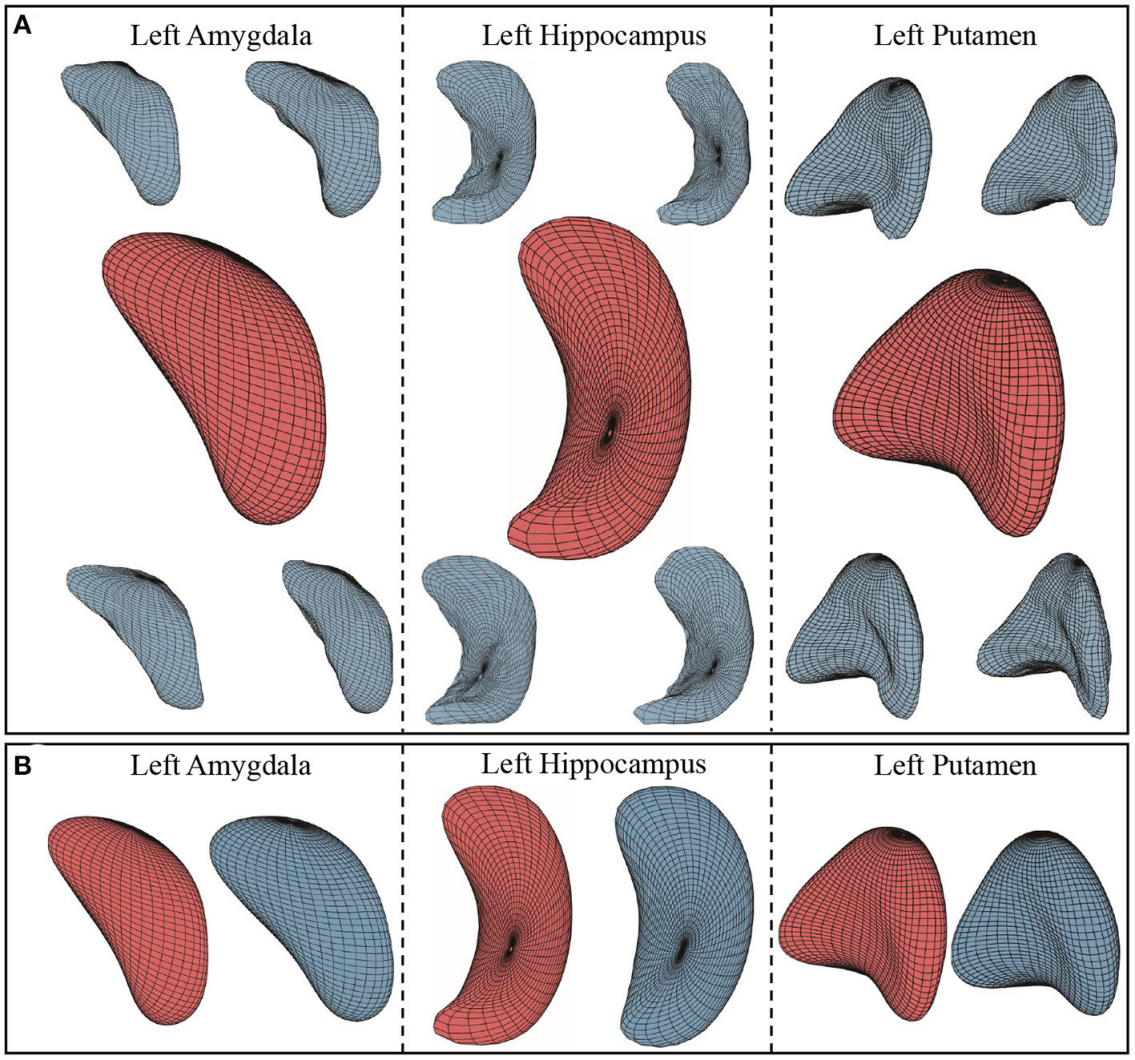
**(A)** Karcher mean surfaces and sample individual surfaces of three subcortical structures. Surrounding blue ones are randomly selected sample individual surfaces, and the middle red ones are Karcher mean surfaces computed using Algorithm 1. **(B)** Comparison between mean surfaces computed with and without elastic registration. Red ones are mean surfaces computed using Algorithm 1 with surface registration, while blue ones are computed without surface registration.

Figure 5 presents the Karcher mean surfaces μ as computed with Algorithm 1 for different structures. Figure 5A shows some registered individual surfaces $f_{i}^{*}$ (drawn around the mean) and their Karcher mean surfaces μ (drawn in the middle). We see that the Karcher mean surfaces capture salient anatomical shape features among the groups while reducing the individual noise. The result of comparison between elastic registered mean surface and unregistered mean surface is presented in Figure 5B. The left red ones are elastic mean surfaces computed with Algorithm 1 and registered surfaces, and the right blue ones are the mean surfaces computed without surface registration. It is observed that elastic mean captures more anatomical shape features, especially obvious at the end part of subcortical structures. On the contrary, some shape information is “averaged out” when computing the mean surface without surface registration. For example, posterior end of the hippocampus surfaces degenerates when computing the mean without elastic registration. As a result, it is more reasonable to use the elastic mean surface for later shape analysis.

##### 2.3.3.3. Shape PCA

Next, we perform Principal Component Analysis (PCA) to capture essential shape variability in a given set of surfaces. We start by computing the covariance matrix *C* for surfaces: $C=\overset{n}{\underset{i=1}{\sum }}V_{i}V_{i}^{T},\;\text{where}\;V_{i}=\text{vec}\left(f_{i}-\mu \right)$, and **vec** denotes vectorization of a matrix. By performing singular value decomposition (SVD) on the covariance matrix *C*, we obtain the left singular vectors as the columns of the unitary matrix *U*. These columns form the principal directions of shape variability the data. The first column is called the 1st principal component, the second column the 2nd principal component, etc. This decomposition also results in singular values that indicate the variance of the shape variability among each of the principal directions.

**Figure 6:**
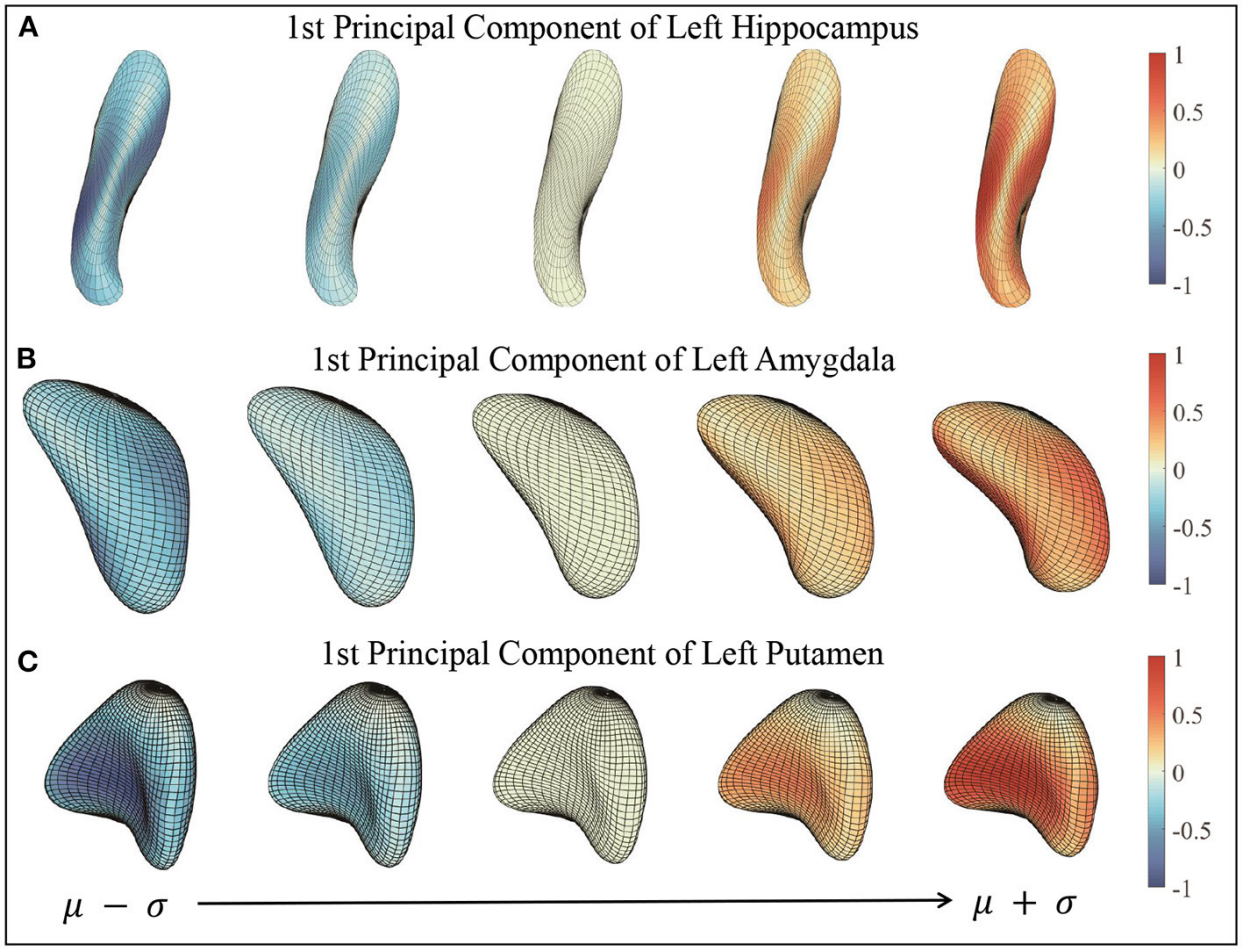
First principal components of three subcortical structures. The figures show the deformation along the path of μ−σ → μ+σ following 1st principal direction. Color indicates the small patch's relative shape difference (deformation level) compared with the mean surface. **(A)** First principal component of left hippocampus. **(B)** First principal component of left amygdala. **(C)** First principal component of left putamen.

Figure 6 illustrates the 1st principal component of left hippocampus, left amygdala, and left putamen surface shape. We show a principal direction using the elastic deformation path μ−σ → μ → μ+σ. Colors on a surface indicate the patch-wise shape differences of that surface when compared with the mean surface. Note that along the 1st principal component: Figure 6A for left hippocampus, the largest shape variability is in the angle of the posterior endings; Figure 6B for left amygdala, the surfaces “bends” more toward the “tail” end; and Figure 6C for left putamen, the curvature of the middle part changes.

The 2nd and 3rd principal components of three subcortical structure surfaces are shown in Supplementary Figures 2–4. We also provide interactive slider graphs to help visualize changes along different principal components for these subcortical structures in the Supplementary material.

##### 2.3.3.4. Low-dimensional shape representations

We use PCA to derive low-dimensional representations of shapes of objects for use in statistical models and regressions. During experiments, we randomly divide all surfaces (of a specific type, say hippocampus) into training and test groups. Then we compute the principal components of shape variation using only the training surfaces and we compute the principal scores for all test surfaces. For a test surface $f_{i}\in F$, *i* = 1, 2, ..., *n* and principal directions *U*(:, *d*), *d* = 1, 2, ..., *n*, the principal score is computed by: *z*_*i, d*_ = 〈*f*_*i*_−μ, *U*(:, *d*)〉. In this way, a high-dimensional object *f*_*i*_ is now represented by a *d*-dimensional vector $z_{i}\in {\mathbb{R}}^{n}$. It is important to note that this representation is invertible. We can map these features back to the object space and reconstruct test surfaces according to: ${\hat{f}}_{i,k}=\mu +\overset{k}{\underset{d=1}{\sum }}z_{i,d}U\left(:,d\right)$. We validate this representation by examining the difference between the reconstructed surface ${\hat{f}}_{i,k}$ and the original surface *f*_*i*_.

**Figure 7:**
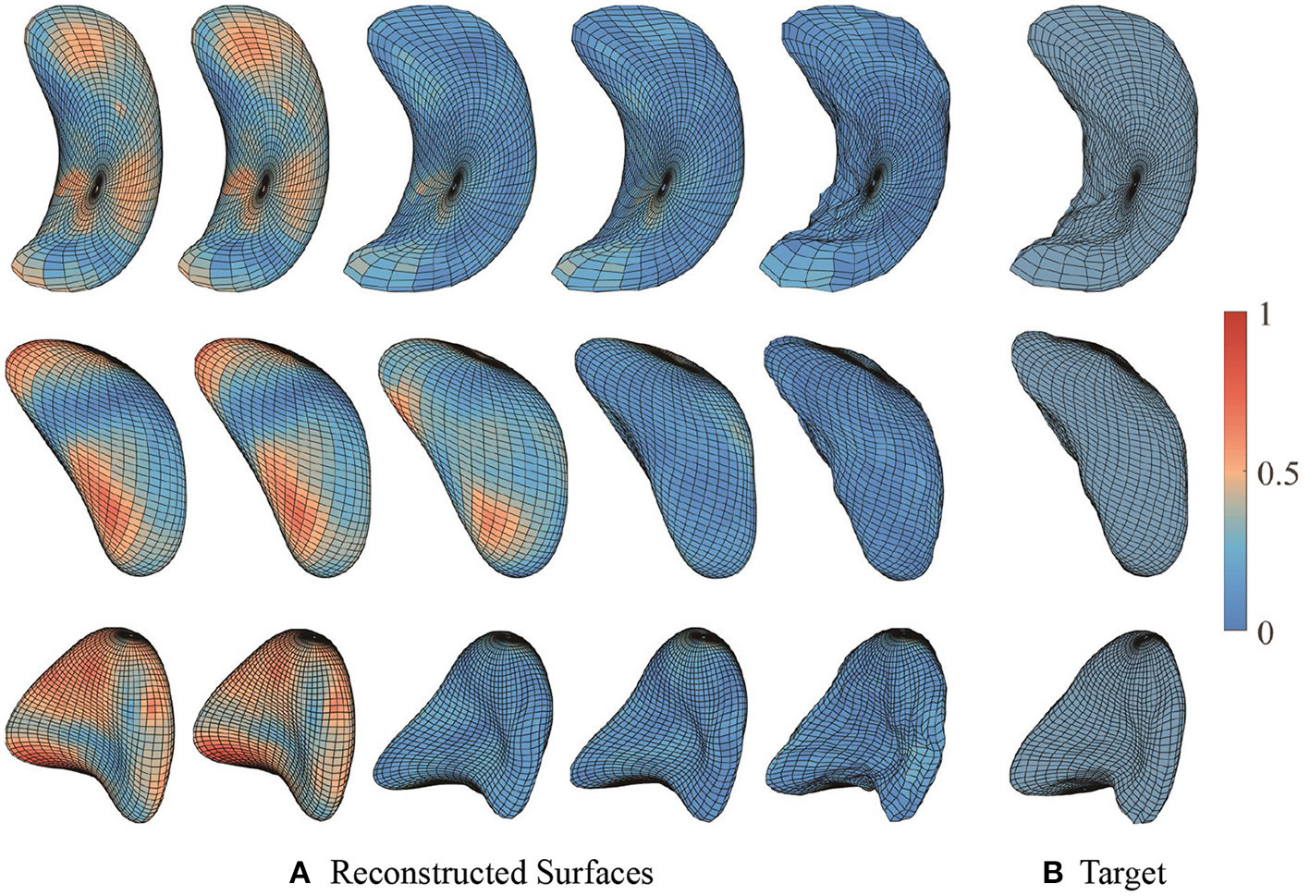
Sample surface reconstructions of three subcortical structures. **(A)** Reconstructed surfaces with first 0, 1, 5, 15, and 72 principal components. **(B)** Target surfaces. Color indicates the small patch's relative shape difference compared with the target surface.

Figure 7 presents some examples of such surface reconstructions. We use 90 × 0.8 = 72 surfaces to compute principal components and the other 18 surfaces to reconstruct and test. The right column shows sample individual surfaces for each subcortical structure, and the left side shows the reconstructed surfaces with *k* = 0, 1, 5, 15, and 72 principal components added to the mean surface respectively. Color indicates the patch-wise relative shape differences between the reconstructed surface and the example surface $\left|f_{i}-{\hat{f}}_{i,k}\right|$. In other words, “1” indicates the largest patch-wise difference along the whole individual surface reconstruction process, and “0” means no reconstruction error in the patch. As more and more principal components added, the patches change color from red to blue, and the shapes of reconstructed surfaces look similar to the sample surfaces. When *k* = 72 principal components (all of the principal components) are used, the reconstructed is almost identical to the original surface. The reconstructed surfaces are mostly blue, which means the difference between reconstructed surface and example surface is relatively very small. This result illustrates that elastic mean and PCA successfully capture the modes of shape variations in subcortical structure surfaces, and represent individual shapes using a small number of PCA coefficients. We provide GIF examples of the surface reconstruction in the Supplementary material.

### 2.4. Comparison with other shape analysis methods

To verify the effectiveness and ability of elastic shape analysis in identifying shape differences attributed to PTSD disease, we compare it with three widely applied shape analysis methods in neuroimaging: vertex-wise analysis, SPHARM-PDM (Styner et al., 2006), and ShapeWorks (Cates et al., 2017).

In order to perform the comparison with vertex-wise analysis, we apply a similar pipeline as our elastic approach and compare the results. We start by representing surfaces using sets of vertices or point clouds. Next, we apply the widespread point cloud registration algorithm, iterative closest point (ICP) (Besl and McKay, 1992), to register individual surfaces. This step is analogous to the elastic registration step. After registration, we compute mean, covariance and PCA, in the same way as elastic shape analysis.

Both the SPHARM-PDM and ShapeWorks are point-based models with open-source software. We generate the comparable results with both methods and compare with elastic shape analysis. We used the suggested software versions and hyperparameters for both methods as follows: **SPHARM-PDM:** Version: 3D Slicer 4.11.20210226 SPHARM-PDM extension (Published on May 26, 2021); Iterations of generating mesh: 500; Subdivision level for linear ikosahedron subdivision: 20; Degree of spherical harmonic expansion: 12; Number of theta iterations: 100; Number of phi iterations: 100. **ShapeWorks:** Version: ShapeWorksStudio 6.2.1; Mesh Grooming: Fill holes; Alignment: Iterative Closest Point; Particle System Parameters: 128 particles, 0.05 initial relative weighting, 1 relative weighting, 1,000 starting regularization, 10 ending regularization, 1,000 iterations per split, 1,000 optimization iterations, 10 normals strength, 10 procrustes interval, 32 multiscale start, and 4 narrow band.

#### 2.4.1. Shape variability explanation

**Figure 8:**
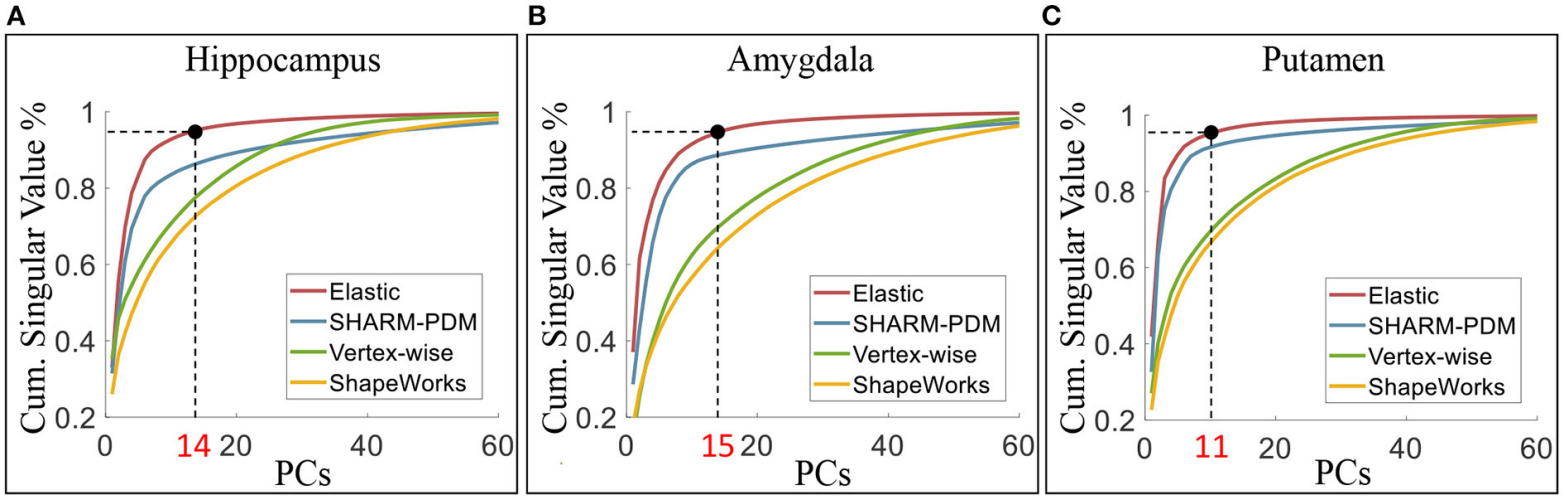
The cumulative proportion of total singular values of the covariance. Under elastic shape analysis, the 1st PCs can explain about 33, 37, and 42% variability of surface shape for left hippocampus **(A)**, amygdala **(B)**, and putamen **(C)**. The cumulative singular values take up more than 95% of total singular values with 14, 15, and 10 PCs, respectively.

We can quantify the level of shape variability explained by the principal components using the cumulative proportion of total singular values, as shown in Figure 8 for the four shape analysis methods. Under elastic shape analysis, the 1st principal component explains about 33, 37, and 42% variability for the left hippocampus, left amygdala, and left putamen, respectively. For these three structures, we can explain over 95% of the variability in shapes with 14, 15, and 10 principal components in total, respectively. Therefore, we will use the first 15 principal components to represent a shape in the subsequent regression analysis. Furthermore, when comparing elastic shape analysis (red lines) with the other three shape analysis methods, we conclude that elastic shape analysis explains more shape variability with the same number of principal components. These results imply that only a small number of PCs can be used to represent the shapes under the proposed elastic shape analysis, which has direct advantages in regression analysis with shape features as elaborated in the sequel.

#### 2.4.2. Low-dimensional representation efficiency

**Figure 9:**
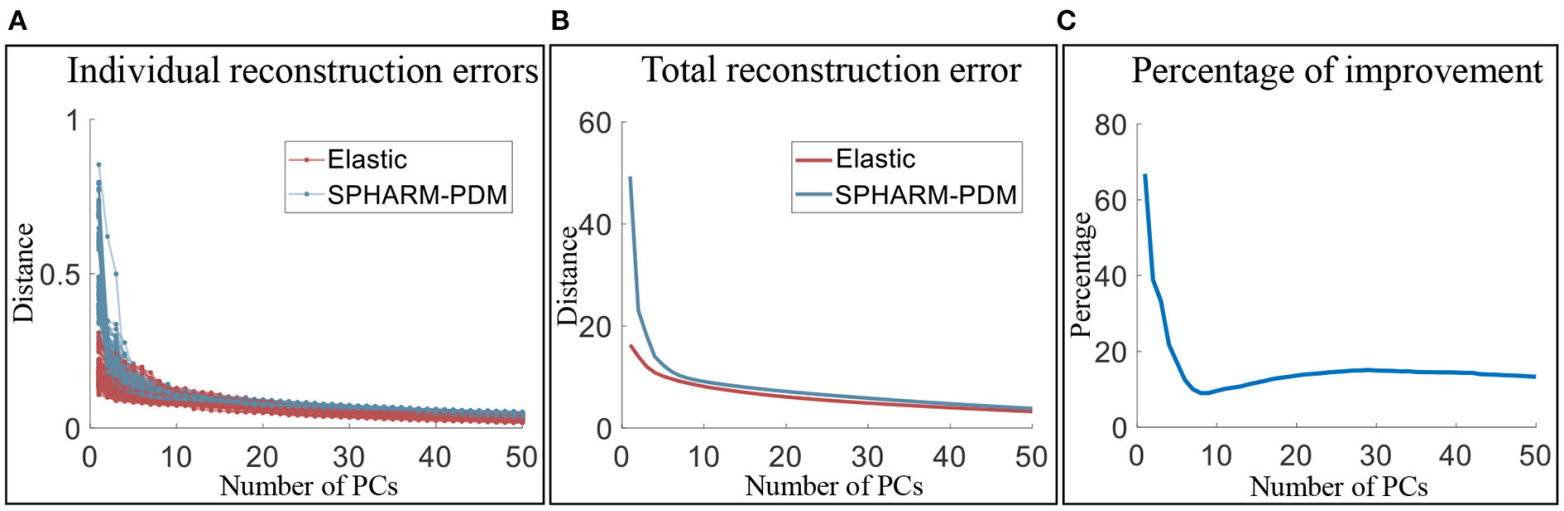
Comparison of the low-dimensional representation efficiency of the elastic shape analysis framework to SPHARM-PDM. **(A)** Individual surface reconstruction errors vs. the number of principal components, **(B)** total reconstruction error of all surfaces vs. the number of principal component, and **(C)** the percentage of efficiency improvement of our elastic shape analysis framework over SPHARM-PDM.

Since SPHARM-PDM performs the second best in explaining the shape variability, we conduct further comparison with SPHARM-PDM to illustrate the necessity of elastic registration and the efficiency of our elastic shape analysis framework bringing the surfaces into low-dimensional representations. We quantify the efficiency of low-dimensional representations using the number of principal components needed for representing shapes up to a fixed reconstruction error. Let *f*_*i*_ be the original surface and ${\hat{f}}_{i,k}$ be the reconstructed surfaces with *k* principal components added. The reconstruction error is defined as $\left||f_{i}-{\hat{f}}_{i,k}|\right|$. Therefore, the framework that generates a smaller reconstruction error with the same fixed *k* is more efficient in low-dimensional shape representation. Figure 9 presents the results for reconstructing surfaces of the three subcortical structures. Figure 9A shows the reconstruction errors of all individual surfaces vs. principal components for the elastic shape analysis framework (red lines) and SPHARM-PDM (blue lines). Figure 9B presents the total distances of all reconstructed surfaces to their original surfaces under different principal components and the results show that elastic shape analysis outperforms SPHARM-PDM in low-dimensional representation efficiency. Figure 9C quantifies this out-performance by the percentage of improvement, indicating that our elastic shape analysis framework has a much superior reconstruction performance when only a few principal components are used to represent the complex shapes.

## 3. Results: Validation of shape analysis using regression modeling

In order to explicitly validate the shape analysis framework, we now conduct regression analysis for modeling PTSD and childhood trauma outcomes based on the low dimensional shape features in the form of principal scores (PS) derived *via* PCA. We fit a series of 5 different linear models with PTSD symptom scales (PSS) as the outcome and another five models with childhood trauma (CTQTOT) as the outcome, listed in Table 1. These models vary with respect to the type of covariates included for analysis. Models 1 and 5 are the most extensive and include age, depression index (BDI), shape features (PS), and interactions between shape features with age and BDI. The interaction terms are all the pairwise interactions between shape features (the first five principal scores for each subcortical structure) and the confounding variables (age and BDI). They are formed by taking the pairwise products of the original predictors, for example, amygdala 1*st* × age, amygdala 1*st* × BDI, hippocampus 1*st* × age, and so on. Here, × denotes the product of two predictors. Models 2 and 6 exclude these interaction terms. Further, models 3 and 7 only include age and BDI, while models 4 and 8 include shape features only. Finally, we also fit models 9 and 10 that augment models 1 and 5, respectively, by including additional covariates in the form of intracranial volume (ICV) that measures the size of the cranium and is an important normalization measure used in morphometric analyses to correct for head size. To capture the shape differences while minimizing noise, we take only the first 15 principal scores (PS) for each surface. These represent the 15 most dominant modes of shape variation to train the model. In these models, we select the most significant predictors using bidirectional stepwise regression. We also focus our shape analysis on including certain subcortical structures in the brain that are known to be associated with PTSD, such as the hippocampus, amygdala, and putamen. Thus, we present the shape analysis results for three groups of surfaces in relation to traumas and PTSD disease.

**Table 1:** Adjusted *R*^2^ of regression models.

**Model no**.	**Model design**	**Adjusted *R*^2^ (%)**
1	PSS ~ Age + BDI + PS + Interactions	63.80
2	PSS ~ Age + BDI + PS	48.26
3	PSS ~ Age + BDI	18.99
4	PSS ~ PS	31.23
5	CTQTOT ~ Age + BDI + PS + Interactions	69.66
6	CTQTOT ~ Age + BDI + PS	38.46
7	CTQTOT ~ Age + BDI	10.97
8	CTQTOT ~ PS	29.25
9	PSS ~ Age + BDI + PS + Interactions + ICV	63.80
10	CTQTOT ~ Age + BDI + PS + Interactions + ICV	69.66

PS, First 15 principal scores; Interactions: age × first 5 PS and BDI × first 5 PS.

### 3.1. Regression models

We use the compact shape representations as predictors in regression models. Specifically, we study the ten linear regression models listed in Table 1. Table 1 shows the adjusted *R*^2^ values for the fitted models, and Tables 2, 3 list the significant principal components with their signs of regression coefficients and the corresponding *p*-values and significant interaction terms for each model.

**Table 2:** Significant principal components.

**Response**	**Predictor**	**Sign of coefficient**	***p*-value**
PSS	Amygdala 4th	+	0.0045
	Amygdala 5th	+	0.0005
	Amygdala 6th	+	0.006
	Hippocampus 2nd	+	0.001
	Putamen 6th	+	0.004
CTQTOT	Amygdala 2nd	+	0.02
	Amygdala 11th	−	0.0008
	Hippocampus 5th	−	0.04
	Putamen 2nd	−	0.01
	Putamen 4th	−	0.03

Predictors are significant under significance level 0.05.

**Table 3:** Significant interactions of model 1 and 5.

**Predictor for PSS**	***p*-value**	**Predictor for CTQTOT**	***p*-value**
Age × Amygdala 4th	0.002	Age × Amygdala 1st	2*10^−5^
BDI × Amygdala 4th	0.005	Age × Amygdala 2nd	0.0007
Age × Hippocampus 3rd	0.0005	Age × Amygdala 4th	3*10^−6^
BDI × Hippocampus 3rd	5*10^−5^	BDI × Amygdala 3rd	7*10^−6^
BDI × Hippocampus 4th	0.002	BDI × Amygdala 4th	1*10^−5^
Age × Putamen 1st	0.001	Age × Hippocampus 4th	0.001
Age × Putamen 2nd	0.005	BDI × Hippocampus 5th	8*10^−5^
Age × Putamen 4th	0.01	Age × Putamen 1st	0.01
Age × Putamen 5th	8*10^−5^	Age × Putamen 2nd	3*10^−5^
BDI × Putamen 1st	0.0007	Age × Putamen 3rd	3*10^−6^
		BDI × Putamen 2nd	0.005
		BDI × Putamen 3rd	0.002
		BDI × Putamen 4th	8*10^−6^

Predictors are significant under significance level 0.01. ×: Product of two variables.

From Table 1, we can see that the models including interactions between shape (PS) and confounding variables (age and BDI) have larger adjusted *R*^2^ value than those excluding interactions. Both Models 1 and 5 achieve large adjusted *R*^2^ values. Although BDI is highly correlated with PTSD symptoms, when we compare Models 2, 4, 6, and 8 with Models 3 and 5 that only includes age and BDI, we reach a higher adjusted *R*^2^. This implies that shape explains more variability in the responses (PSS and CTQTOT) than BDI. Next, we focus on the significant shape principal components of each subcortical structure.

From Table 2, we see that PTSD symptoms are most correlated with subcortical shapes of the following principal components: amygdala 4th, amygdala 5th, amygdala 6th, hippocampus 2nd and putamen 6th. Similarly, the most significant subcortical shape changes associated with childhood traumatic experience lie in the principal directions: amygdala 2nd, amygdala 11th, hippocampus 5th, putamen 2nd, and putamen 4th. After controlling for ICV, the principal shape components of elastic shape analysis are still found to be statistically significant. This result indicates that the elastic registration procedure is sound and can naturally control for the variability in the head size after registration. Clearly, the elastic shape analysis approach can successfully register the different brains, so that variable head sizes do not impact the analysis and do not provide any additional gains in explaining the variability in the clinical outcomes. Table 3 presents the significant interactions between shape and confounding patterns correlated with PTSD and traumatic experience.

### 3.2. Shape pattern

**Figure 10:**
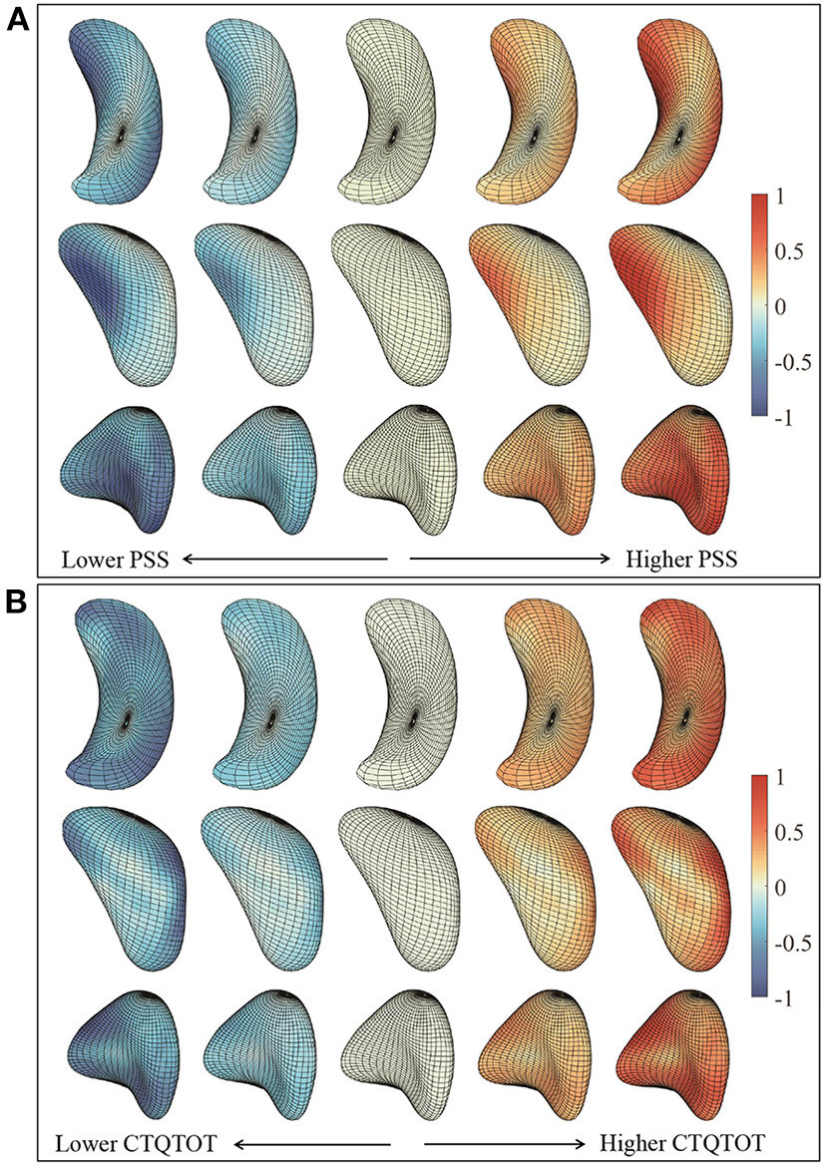
**(A)** Shape deformation along the most significant principal components on PTSD symptom scale. The surfaces to the left have less severe or no PTSD symptoms, and the surfaces to the right have more severe PTSD symptoms. **(B)** Shape deformation along the most significant principal components on childhood traumatic experience inventory. The surfaces to the left have less or no childhood traumatic experience, and the surfaces to the right have more childhood traumatic experience. Color indicates the small patch's relative shape difference along the direction.

In order to understand variations associated with different PSS and CTQTOT levels, we visualize the significant principal components for each subcortical structure. From Figure 10A, we observe that with severe PTSD symptoms:

- Left hippocampus surface moves along the positive direction of 2nd principal component, which shows a shrunken anterior end and curved body part;
- Moving along the positive direction of 4th, 5th, and 6th principal components, the left amygdala surface mainly deforms at the “head” end, where the central nucleus lies. The “head” end tends to indent;
- Left putamen surface's concave middle part has a larger curvature, and the end part gets thinner and sharper along the positive direction of the 6th principal component.

Figure 10B shows that with severe childhood trauma:

- Left hippocampus surface has thinner anterior and posterior ends moving along the negative direction of 5th principal component;
- Left amygdala surface has shrunken head end and the left side appears more flattened;
- Left putamen surface's middle part is hollower and the end part gets sharper along the negative direction of 2nd and 4th principal component.

Through these visualizations, we find that the shape changes of three subcortical structures following different levels of PTSD symptoms and childhood traumatic experience are consistent. This, in turn, supports the results of regression models. Subjects with more childhood traumatic experiences potentially have more severe PTSD, and the shape of subcortical structures deforms in the same direction.

Additional displays of deformations along the most significant principal components for PSS and CTQTOT are presented in Supplementary Figures 5, 6. The interactive graphs to visualize shapes at different levels of PSS and CTQTOT are also presented in the Supplementary material.

### 3.3. Result comparisons

To compare the four methods in the regression context, we replace the elastic shape analysis principal scores with the principal scores of other shape analysis methods and repeat the experiments with the first eight model designs. Table 4 lists the model adjusted *R*^2^ values of the four methods. Regression models trained with elastic shape analysis principal scores outperform those trained with the principal scores of other shape analysis methods in all six models (note that models 3 and 7 are independent of principal scores, so the adjusted *R*^2^ values of these two models are identical for all four methods). Besides, with the same number of principal components, those computed with the elastic shape analysis method contain more shape information (shape variation) because of the geometric properties discussed in Section 2.

**Table 4:** Comparison: adjusted *R*^2^ values for models under four methods.

**Model no**.	**Vertex-**	**SPHARM-**	**ShapeWorks**	**Elastic**
	**wise (%)**	**PDM (%)**	**(%)**	**shape (%)**
1	58.96	59.41	50.92	**63.80**
2	46.55	40.69	34.34	**48.26**
**3**	18.99	18.99	18.99	**18.99**
4	23.32	31.20	24.23	**31.23**
5	55.63	54.20	23.97	**69.66**
6	24.35	24.37	32.92	**38.46**
**7**	10.97	10.97	10.97	**10.97**
8	18.10	22.52	28.09	**29.25**

The bold values indicate highlights for the results of the article.

From these results, we conclude that elastic shape analysis is more effective and accurate in identifying the shape differences of subcortical structures correlated with PTSD and childhood traumatic experience, when compared to other widely applied shape analysis methods.

## 4. Discussion

We have proposed a comprehensive shape-analysis approach that treats the brain structures as continuous surfaces instead of a collection of discrete points. One of the key aspects of the approach is that it incorporates crucial registration steps such as rigid motions, global scaling, and parameterizations of surfaces in a unified way. It uses a novel SRNF representation and an elastic metric that appropriately measures geodesic distances between shapes while registering them. Incorporating these important registration steps when comparing shapes helps reduce errors due to additional pre-processing and registration steps that are routinely employed in existing shape analysis approaches and helps enhance the accuracy of the proposed method.

Another important feature of the proposed approach is that it registers shapes by pairwise deformations and comparisons, and it does not need a standardized brain template for registration and shape analysis. This is a useful feature that is not present in existing shape analysis methods that usually employ a standardized brain template for registration and subsequent shape analysis (for example, Klein et al., 2009). In addition, the proposed approach is able to compute a standardized template that represents the average shape of the sample of images in the data *via* the Karcher mean, and is further able to provide confidence intervals around this Karcher mean that provides measures of uncertainty corresponding to the brain shapes in the population.

Our analysis of the MRI neuroimaging data for trauma-exposed participants illustrated that the proposed approach was able to capture more than 95% of the variability in subcortical shapes using a moderate number of principal components, whereas a considerably higher number of components were needed to capture similar levels of variability under other alternate widespread shape analysis methods. We conclude therefore that the elastic shape analysis comprises a more parsimonious characterization of the shape of subcortical brain regions. This provides a benefit for a number of modeling techniques that would benefit from sparser representation of the neural features of interest.

These principal components were then used as shape features to predict continuous clinical measures in PTSD in conjunction with additional exposure variables such as trauma and their interactions. This joint analysis is a significant advantage over the other shape analysis methods, where such interactions are challenging to include because of an inflated number of model parameters. The predictive analysis yielded high adjusted *R*^2^ values that were considerably higher than what is typically observed in the psychiatric neuroimaging literature. We were able to explain a unique 29% of the variance in PTSD symptom severity using the principal scores, above and beyond effects of age or depressive symptoms. In contrast, large collaborative meta-analyses of PTSD neuroimaging biomarkers find small effect sizes ranging from *d* = 0.06−0.17 across subcortical volumes, major white matter tracts, and regional cortical thickness (Logue et al., 2018; Dennis et al., 2019; Wang et al., 2020), and are often not able to co-vary for potential comorbid conditions.

Furthermore, in comparison with the other medical shape analysis approaches, elastic shape analysis produced increases in sensitivity for the association with PTSD symptoms and the association with childhood trauma exposure. The current findings suggest that these minor effects may arise in part from methodological issues with signal detection and precision in post-acquisition analysis of the images. This is encouraging and suggests that precision psychiatric biomarkers may become more feasible and translatable with additional development of analytic tools for characterizing brain structural alterations. We must also acknowledge, however, the major role that heterogeneity in patient populations and symptom presentation play in moderating effect sizes in PTSD. We conjecture that the increased sensitivity observed under elastic shape analysis is due to the incorporation of accurate shape features (principal components), along with the subsequent capacity to incorporate supplementary variables and their interactions in our analysis.

We identified an association between PTSD symptom severity and complex alterations in the hippocampus, amygdala, and putamen. With increasing PTSD symptom severity, the left hippocampus showed shrinkage of the medial wall of the head as well as lateral aspects of the tail, producing a more curved body shape. Although elastic shape analysis is not designed to investigate specific subfields of the hippocampus, this could be consistent with the location of CA1 and/or subiculum along the longitudinal axis of the hippocampus, consistent with prior work (Chen et al., 2018; Bae et al., 2020). The left amygdala showed an indentation in superior aspects located near central subnuclei. This differs from prior literature in male veterans showing either no shape differences (Bae et al., 2020), or larger central and medial nuclei (Klaming et al., 2019; Morey et al., 2020). In contrast, our study was conducted in women exposed to civilian traumas such as interpersonal violence. Lastly, the left putamen showed greater concavity with thinning and sharpening of the medial end, near the nucleus accumbens. The link with brain regions involved in motivation and reward is interesting given the affective and motivational blunting observed in the numbing symptoms of PTSD, although striatal morphology has received very little attention in studies of trauma and PTSD.

The utility of the principal components as shape features are provided *via* a Matlab GUI interactive tool that enables one to visualize how the brain shape changes as the first few principal components are varied (provided in Supplementary material). Such a tool provides a novel way to visualize changes in the brain shape that is expected to have an important impact for investigators.

## 5. Conclusions

This study uses the elastic shape analysis to compute shape summaries (mean, covariance, PCA) of subcortical data from the Grady Trauma Project (GTP). Having obtained PCA-based low-dimensional representation of shapes, we build regression models to predict PTSD clinical measures that use shapes of hippocampus, amygdala, and putamen as predictors and have considerably great predictive power. Furthermore, we localize and visualize the subcortical shape deformations related to change in PTSD severity. This tool can also provide physicians and clinicians a novel way to visualize localized changes or deformations in the brain anatomy for statistically significant shape features or principal components. Prospective studies can be carried out in larger sample/data sizes and involving additional subcortical structures to improve predictions of PTSD clinical measures. In addition, some more computationally efficient algorithms can improve the proposed approach.

## Data availability statement

The data analyzed in this study is subject to the following licenses/restrictions: due to privacy and ethical considerations, the data cannot be made openly available at this stage. However, the data will be shared upon reasonable request. Requests to access these datasets should be directed to JS, jennifer.stevens@emory.edu.

## Ethics statement

The studies involving human participants were reviewed and approved by Emory University. The patients/participants provided their written informed consent to participate in this study.

## Author contributions

JS and NF organized the dataset. YW, SK, and AS contributed to the design of the study. YW performed the statistical analysis and wrote the first draft of the manuscript. SK, JS, and AS wrote sections of the manuscript. All authors contributed to manuscript revision, read, and approved the submitted version.

## Funding

This research was conducted in part with support from NIH R01 MH120299 and NSF DMS 1953087.

## Conflict of interest

The authors declare that the research was conducted in the absence of any commercial or financial relationships that could be construed as a potential conflict of interest.

## Publisher's note

All claims expressed in this article are solely those of the authors and do not necessarily represent those of their affiliated organizations, or those of the publisher, the editors and the reviewers. Any product that may be evaluated in this article, or claim that may be made by its manufacturer, is not guaranteed or endorsed by the publisher.
