# Supplementary material for: Elastic shape analysis of brain structures for predictive modeling of PTSD
Source: Front Neurosci. 2022 Sep 1;16:954055. doi: 10.3389/fnins.2022.954055 (PMC9475197; doi:10.3389/fnins.2022.954055)
Supplement: Supplementary file 1 [file Data_Sheet_1.pdf]

Supplementary Material

1 SUPPLEMENTARY FIGURES

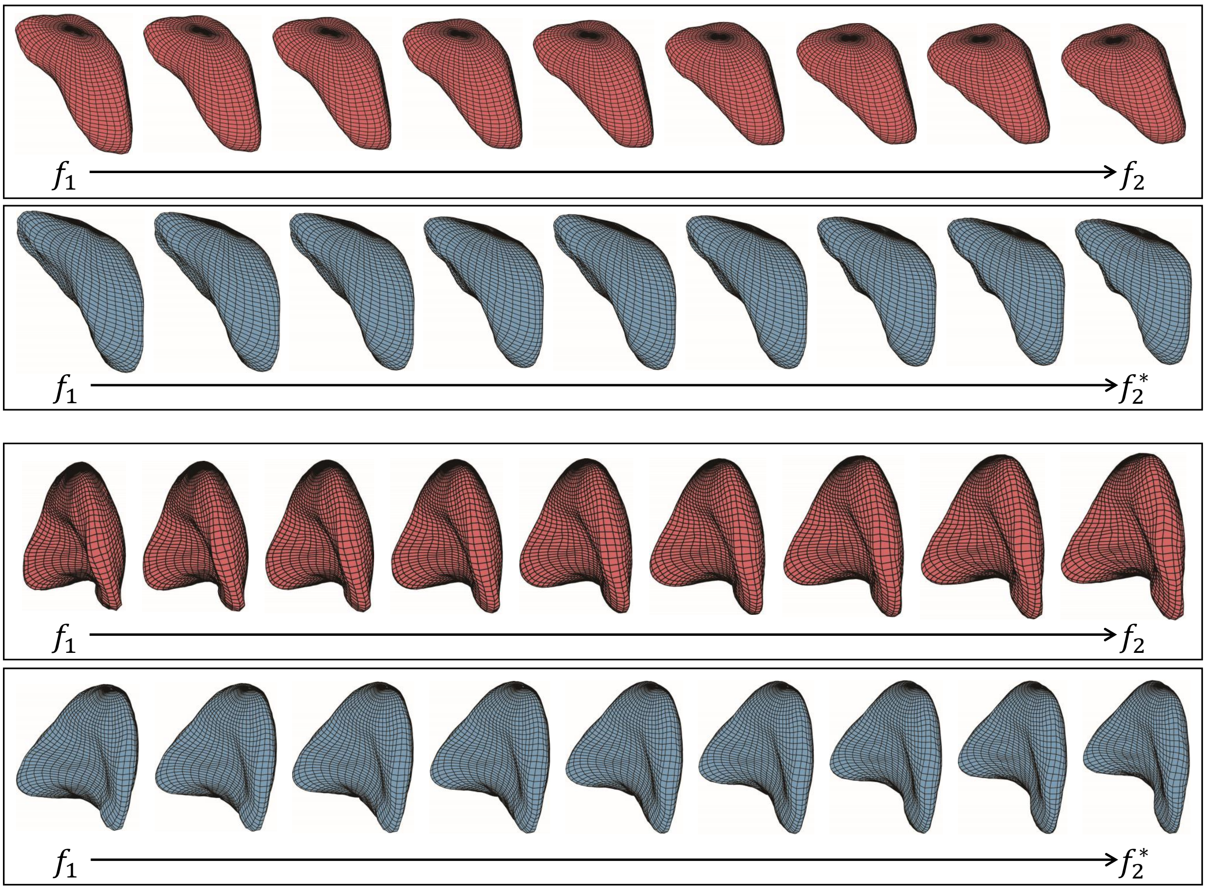

Figure S1. Examples of geodesic between left amygdala and left putamen surfaces. Red ones show the geodesics between unregistered surfaces, and blue ones show the geodesics between surfaces that are elastic registered.

2 SUPPLEMENTARY VIDEOS AND APPLICATIONS

The GIF videos, interactive slider graphs, and Matlab GUI interactive tools are available online in the Dropbox folder.

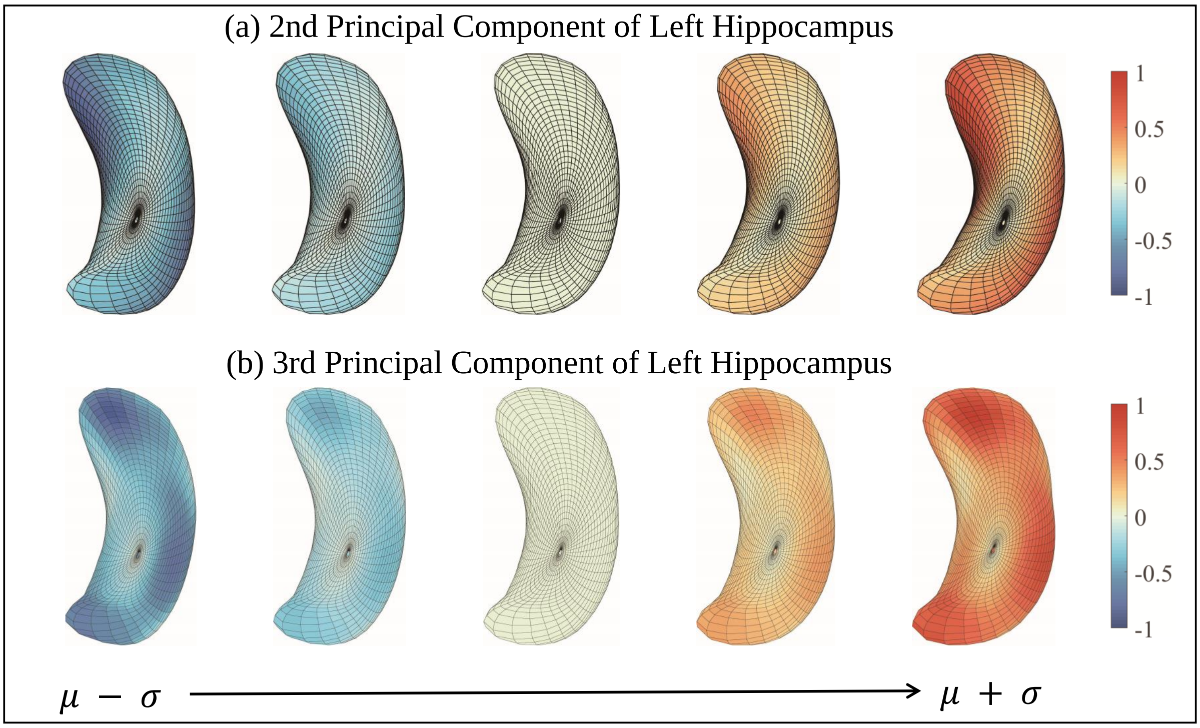

Figure S2. 2nd and 3rd principal components of left hippocampus surfaces. (a) following the positive direction of 2nd principal component, the anterior end of hippocampus surfaces tends to shrink; (b) the anterior end of hippocampus gets flatten and the posterior end swells following the positive 3rd principal direction.

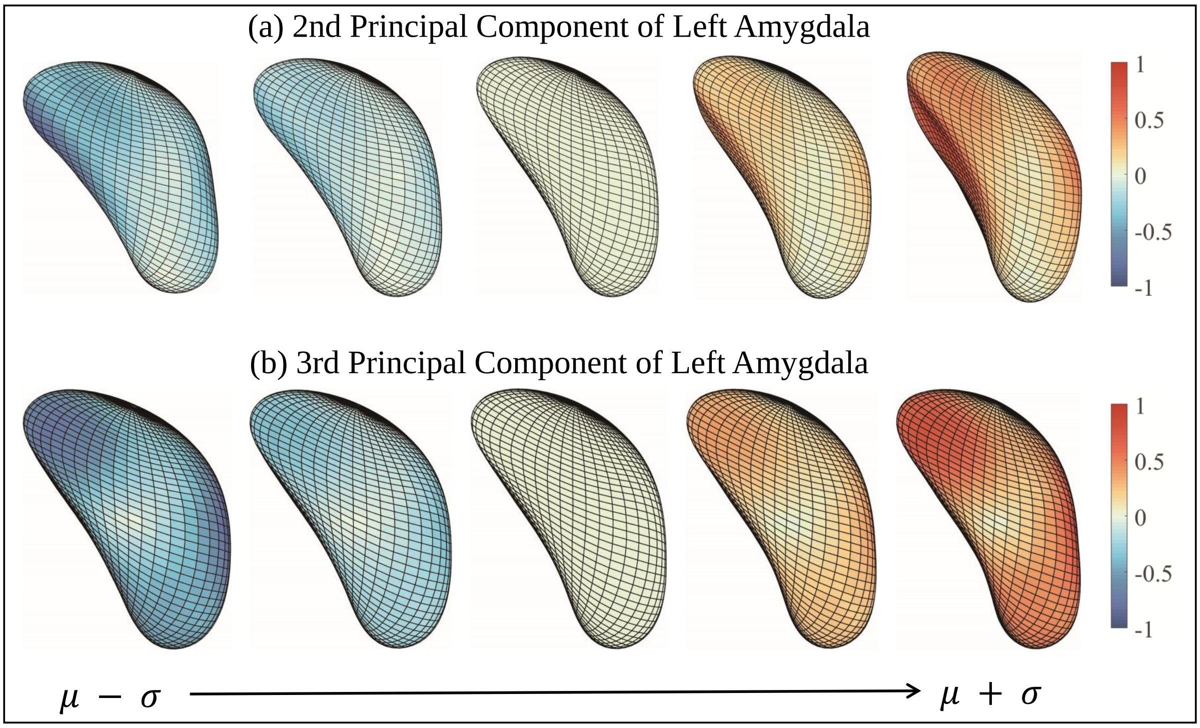

Figure S3. 2nd and 3rd principal components of left amygdala surfaces. (a) the angle and size of the ”head” end changes along the 2nd principal component; (b) amygdala surface gets stretched to be thinner and longer following the positive direction of 3rd principal component.

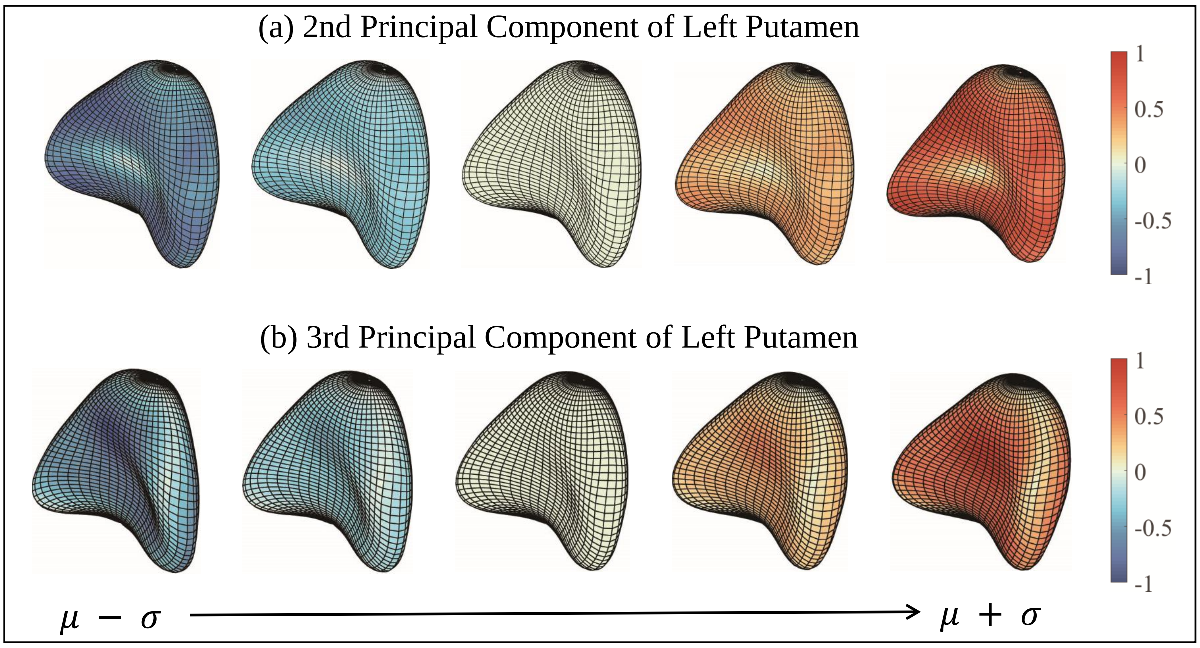

Figure S4. 2nd and 3rd principal components of left putamen surfaces. (a) two ends head to opposite directions and become closer on positive 2nd principal direction; (b) the anterior end stretches out following the positive direction of 3rd principal component.

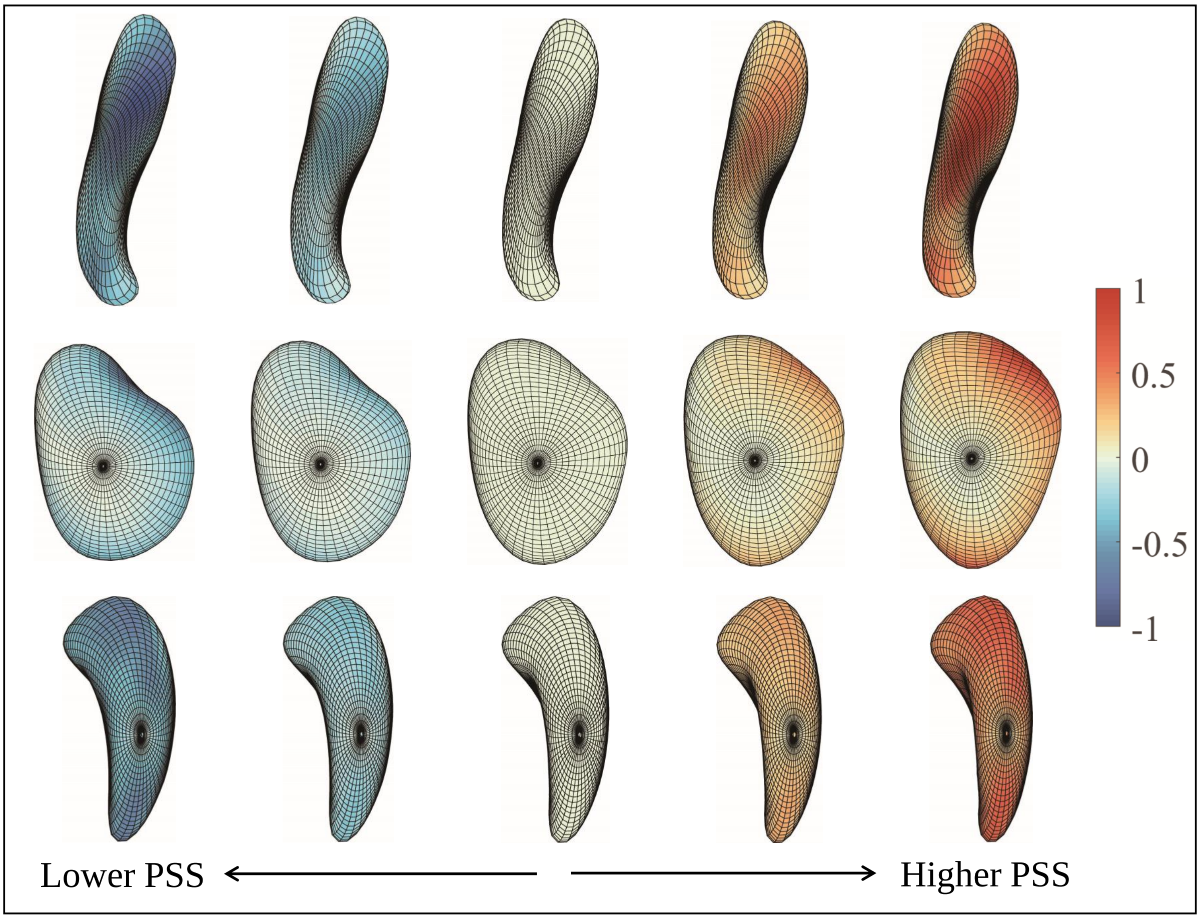

Figure S5. Another view for the shape deformation along the most significant principal components on PTSD symptom scale. The surfaces to the left have less severe or no PTSD symptoms, and the surfaces to the right have more severe PTSD symptoms. Color indicates the small patch's relative shape difference (deformation level) along the direction.

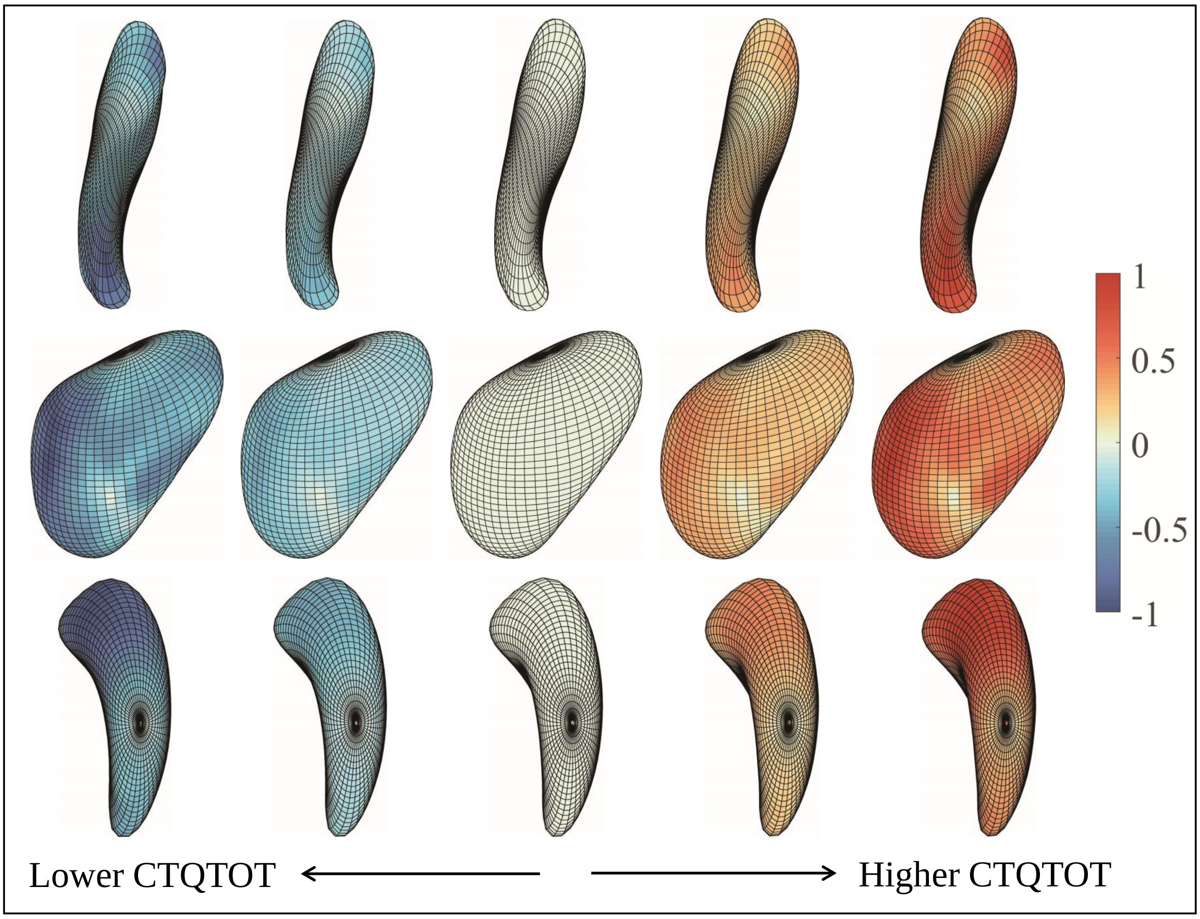

Figure S6. Another view for the shape deformation along the most significant principal components on childhood traumatic experience inventory. The surfaces to the left have less or no childhood traumatic experience, and the surfaces to the right have more childhood traumatic experience. Color indicates the small patch's relative shape difference (deformation level) along the direction.
